# Systematic review and meta-analysis of iodine nutrition in modern vegan and vegetarian diets

**DOI:** 10.1017/S000711452300051X

**Published:** 2023-11-14

**Authors:** Elizabeth Rose Eveleigh, Lisa Coneyworth, Simon J. M. Welham

**Affiliations:** Division of Food, Nutrition and Dietetics, School of Biosciences, The University of Nottingham, Sutton Bonington LE12 5RD, UK

**Keywords:** Iodine, Iodine intake, Micronutrients, Vegan, Vegetarian

## Abstract

Vegan and vegetarian diets are widely supported and adopted, but individuals following such diets remain at greater risk of iodine deficiency. This systematic review and meta-analysis was conducted to assess the iodine intake and status in adults following a vegan or vegetarian diet in the modern day. A systematic review and quality assessment were conducted from October 2020 to December 2022 according to Preferred Reporting Items for Systematic Reviews and Meta-Analyses (PRISMA) and Meta-analysis of Observational Studies in Epidemiology (MOOSE) guidance. Studies were identified in Ovid MEDLINE, Web of Science, PubMed, and Scopus. Eleven articles were eligible for review containing 4421 adults (aged ≥ 18 years). Vegan groups had the lowest median urinary iodine concentration (mUIC) (12·2/l). None of the dietary groups had mUIC within the optimal range for iodine status (100–200 µg/l) (WHO). Vegan diets had the poorest iodine intake (17·3 µg/d) and were strongly associated with lower iodine intake (*P* = < 0·001) compared with omnivorous diets. Lower intake in vegan diets was influenced by sex (*P* = 0·007), the presence of voluntary or absence of Universal Salt Iodisation (USI) programmes (*P* = 0·01 & *P* = < 0·001), and living in a country with adequate iodine nutrition (*P* = < 0·001). Vegetarians and particularly vegans living in countries with no current USI programme continue to have increased risk of low iodine status, iodine deficiency and inadequate iodine intake. Further research into the usefulness of mandatory fortification of vegan appropriate foods is required.

Environmentally sustainable diets, that are rich in plant foods, are advocated as a solution to feeding the growing human population and reducing the impact of our food systems on the planet^(1)^. Today, vegetarian and vegan diets are widely supported and adopted throughout the population, particularly among young people and women^(2–4)^. Vegans do not consume any animal products, whereas vegetarians exclude only meat and fish^(5)^. The inclusion of fish in a vegetarian diet is often called a pescatarian diet^(5)^. There are also growing numbers of people in Western countries reducing their animal product consumption who define as flexitarian^(5,6)^.

The rising interest in plant-based eating has increased demand for a diversity of commercially available alternative products (e.g. plant-based milk) suitable for vegetarians, vegans or those wishing to reduce their intake of animal products^(7)^. Fast-food chains have similarly designed animal-free products targeting consumers^(7,8)^, and restaurants have expanded menu options to suit all dietary requirements^(7,8)^. Individuals now have a more diverse pool of foods suitable for their dietary preferences than before, permitting huge variations in food and nutrient intake. However, although the breadth of choice is beneficial for diet diversity, there continue to be difficulties in achieving adequate quantities of some micronutrients, most notably iodine^(9)^.

Iodine is essential for the synthesis of the thyroid hormones (triiodothyronine (T3) and thyroxine (T4)) and is dependent on adequate supply via the diet^(10)^. The WHO recommends a daily intake of 150 μg/d for adults (18+ years) and 250 μg/d for pregnant and lactating women to maintain thyroid hormones synthesis and prevent the development of associated ‘iodine deficiency disorders’ (IDD)^(10,11)^. However, iodine recommendations are often country-specific, in the UK a Reference Nutrient Intake (RNI) of 140 µg/d is advised for adults (19–50 years)^(12)^. The UK currently has no RNI set for pregnant/lactating women^(12)^. The thyroid hormones have a vital role in the regulation of metabolism, growth and fetal neurological development^(13–15)^. IDD include hypothyroidism, goitre, the formation of thyroid nodules and cretinism (as a consequence of gestational exposure)^(10,14,16)^. Iodine excess (consumption of > 1000 µg/d iodine) can also have health repercussions, including hypo- and hyperthyroidism^(17,18)^.

Iodine deficiency remains a public health issue worldwide^(19)^, although the number of countries with adequate iodine intake has nearly doubled over the past 20 years^(20)^. Improvements in global iodine intake and status have largely been due to the effective implementation of universal salt iodisation (USI)^(19,20)^. However, not all countries currently facilitate USI programmes, and other factors (e.g. low household coverage, low usage, low or no accessibility, and inadequate quality) may limit its effectiveness^(19)^. Currently in industrialised countries, the predominant dietary iodine sources are iodised salt, cows′ milk, dairy products, fish and seafood, eggs and fortified foods (e.g. bread)^(21)^. Plant foods are generally low in iodine, unless bio-fortified, with seaweed being an exception^(22,23)^. The iodine content of seaweed is highly variable and can be linked to iodine excess in certain populations^(24–27)^.

Individuals who restrict iodine-rich foods from their diet or are solely dependent on iodine provision from plant foods are at risk of iodine deficiency^(28)^. The increased risk of iodine deficiency in vegans and vegetarians was highlighted by the Scientific Advisory Committee on Nutrition (SACN) in 2014^(29)^. Since its publication, several studies have assessed iodine nutrition to be poorer in these groups^(4,30–34)^. Innovation in the vegan and vegetarian food sector has resulted in a rapid proliferation of products. Many items, such as meat alternatives, are not often fortified with iodine^(35,36)^. Alternative dairy products such as alternative ‘milks’ and ‘yogurts’ are now regularly fortified with vitamin B_12_, D and Ca^(36–40)^, but rarely with iodine^(36,37,39)^. Additionally, iodine supplements are still not universally consumed by individuals following vegan and vegetarian diets^(34)^.

We previously reviewed the literature on iodine intake and the status of vegans and vegetarians living in industrialised countries in 2020^(28)^. Our study concluded that those individuals’ not consuming seaweed or iodine-containing supplements were at risk of poor iodine nutrition. However, half of the studies included in our review were published before meat-free diets entered the mainstream in many countries (2010) and, therefore, may not represent the diversity of ‘modern-day’ vegan and vegetarian diets observed in today’s society^(28)^. In the past, traditional vegan diets were reliant on the consumption of wholegrains, pulses, fruit and vegetables^(9)^. Whereas, modern-day vegan dietary patterns conflict with traditional vegan dietary patterns due to the improved availability of foods that are often designed for convenience and are ultra-processed (e.g. snacks foods, alternative milks and meats, etc.)^(9)^. Greater food availability and choice have permitted a larger number of dietary patterns to be followed within the standard definitions of vegan and vegetarian^(9)^.

Following the publication of our review, many new studies have examined iodine in the diet of vegans and vegetarians. Iodine deficiency is still a major public health issue and given that sustainable diets are being promoted in many countries worldwide, a re-evaluation of the literature is needed to monitor iodine nutrition and deficiency in those who select to follow a vegan or vegetarian diet in the modern day.

This review aims to assess the iodine intake and status in adults following a vegan or vegetarian diet in the modern day. The objectives included (1) determination of the iodine intake and food consumption in vegan and vegetarian adults; (2) assessment of the iodine status and prevalence of iodine deficiency using urinary iodine concentration (UIC); (3) comparison of the iodine intake, status, and prevalence of deficiency between modern-day vegans, vegetarians, and omnivores; and (4) completion of a meta-analysis to provide a more precise estimate of the effect size results of individual studies. We hypothesise that vegans and vegetarians will continue to be a subgroup at risk of iodine deficiency; however, given the increase in the availability of iodine fortified foods suitable for individuals following these diets, iodine deficiency may be less severe.

## Methods

### Search strategy

The Preferred Reporting Items for Systematic Reviews and Meta-Analyses (PRISMA) and the Meta-analyses of Observational Studies (MOOSE) checklist were used to complete this systematic review^(41,42)^. A systematic search of the literature was performed from 5 October 2020 to 16 December 2022. Electronic databases (Ovid MEDLINE, Web of Science, PubMed and Scopus) were explored. Searches were completed with appropriate text terms with truncation and medical subject headings. Our search strategy is the same as that described in our previous systematic review^(28)^ (online Supplementary Table 1). All database searches were refined by ‘humans, adults (aged > 18 years), English language, full-papers, and publications between January 2020–December 2022’. Search lists were exported into EndNote^TM^ before the removal of duplicates. Additional studies were identified via reference lists of relevant published materials and citation searching. A review protocol has not been published prior to our literature search.

### Population and outcomes

The population–intervention–comparison–outcome (PICOS) formulation was used to assess study eligibility (Table 1)^(43)^.

**Table 1.** Population–intervention–comparison–outcome (PICOS) criteria for study inclusion and exclusion^(43)^

Criteria category	Inclusion	Exclusion
Population	Adults (aged ≥ 18 years)	Individuals (aged < 18 years), unless results display separate data; adults residing in developing countries; populations with a high prevalence of thyroid disorders
Intervention/exposure	Participants with any type of dietary preference or restriction. Voluntary or otherwise	Use of a dietary grouping without defining diet characteristics
Comparators	Differing dietary preference or restriction	None
Outcome measure	Iodine intake or status measured by urinary iodine concentration (UIC) or analysis of dietary records	No analysis of iodine intake or status; use of thyroid measures alone for iodine intake and status
Study design	Any study design with relevant outcomes	None

### Data extraction

Extraction forms identified study information including author name, journal, publication date, study location, participant characteristics, dietary groups addressed and grouping method, relevant outcome measures (length of diet, iodine intake, status and supplement use), and any key findings or study considerations (limitations, etc.). Where possible, gendered data were considered separately. Data extraction was completed by the first author.

### Classification of outcomes

The nomenclature defining dietary preferences can vary; therefore, dietary groups were compared according to the following classifications (Table 2). We also recorded the method of dietary grouping used and the length of dietary adherence for each study. For articles where individuals are following vegan/vegetarian/etc. diets as part of an intervention, post-intervention data were used.

**Table 2.** Definition of common vegetarian and vegan diet types

Type	Definition
Omnivore*	Includes meat, poultry, fish, eggs and dairy products
Flexitarian†	Occasionally excludes meat and poultry but remains consumption of fish, eggs and dairy products
Pescatarian†	Excludes meat and poultry but remains consumption of fish, eggs and dairy products
Lacto-ovo vegetarian†	Excludes meat, poultry and fish but remains consumption of dairy products and eggs
Lacto vegetarian†	Excludes meat, poultry, fish and eggs but remains consumption of dairy products
Ovo vegetarian†	Excludes meat, poultry, fish and dairy products but remains consumption of eggs
Vegan†	Excludes all animal products (meat, poultry, fish, eggs and dairy products)
Strict raw‡	Excludes all animal products (meat, poultry, fish, eggs and dairy products) and heated food items.

* Diet definition includes descriptions of omnivore (basic) and omnivore (regular) from Kowalska *et al.* 2020^(57)^. Omnivore (basic) includes all food groups including meat, poultry, fish, dairy products and eggs; omnivore (regular) describes data collected from 24-h dietary recalls from ten Polish adults that were used to make standard ‘menus’ for omnivorous participants.

† Diet definitions from Phillips 2005^(5)^.

‡ Diet definition from Abraham *et al*. 2022^(53)^.

The WHO criteria were used to assess iodine status using UIC^(11,44)^. Therefore, a median UIC > 100 μg/l was considered ‘sufficient’ and a median UIC < 100 μg/l was considered ‘deficient’, with severity of IDD based on UIC being: 50–99 μg/l mild, 20–49 μg/l moderate and > 20 μg/l severe^(11,44)^. Recommended adequate iodine intake varies across countries, so the WHO RDA for adults and adolescents of 150 μg/d was used to assess iodine intake between studies^(11,44)^.

### Quality assessment

The quality of observational cohort, cross-sectional and case–control (matched pairs) studies was assessed using the Newcastle–Ottawa scale (good, fair or poor)^(45)^. The quality of modelling studies was not assessed (*n* 1). Quality assessment was completed by the first author and was reviewed by the study team (online Supplementary Table 2).

#### Statistical analysis

We performed meta-analysis and subgroup analysis to compare iodine intake and status in vegan and vegetarian diets compared with omnivores using the software RevMan (version 5) designed by Cochrane^(46)^. We assumed that the studies are heterogenous; therefore, we selected a random effects mode. For studies reporting data as medians (IQR), we estimated the sample mean and standard deviation using calculations as described by Wan *et al* ^(47)^.We separated comparisons of vegan and vegetarian diets into different forest plots to avoid arbitrary omission of relevant groups and double counting of participants. If the test for overall effect were *P* = < 0·05, the following covariables for subgroup analysis for both meta-analyses were used: sex (female *v.* mixed sex), USI status and iodine status by country. Countries were categorised into iodine-deficient or -sufficient according to the Iodine Global Network (IGN) Scorecard^(20)^.

## Results

**Fig. 1.**
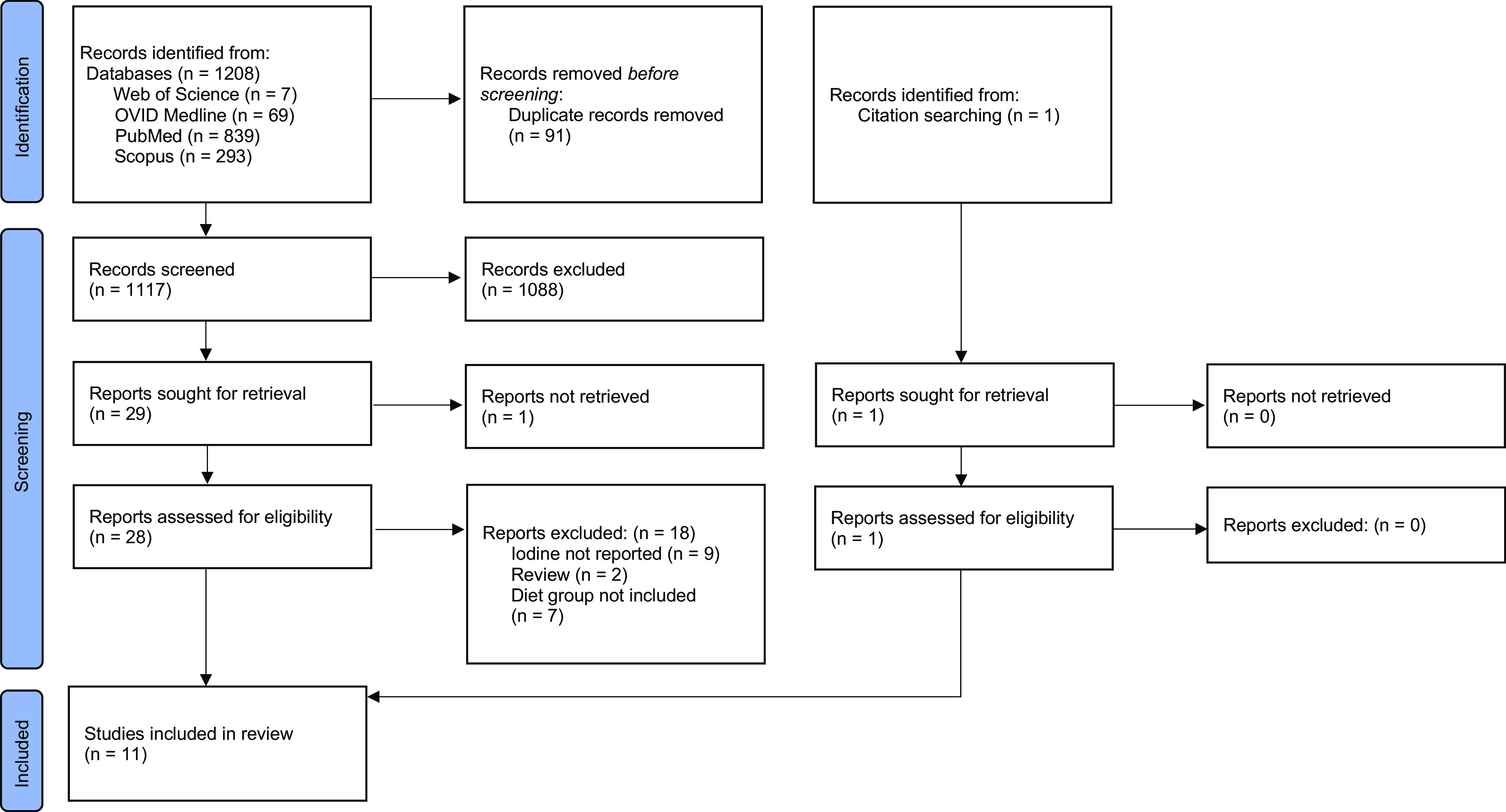
PRISMA 2020 flow diagram of the study selection process^(48)^. PRISMA, Preferred Reporting Items for Systematic Reviews and Meta-Analyses.

The initial literature search yielded 1208 articles. Following screening and exclusion, twenty-eight reports were assessed for eligibility. The PRISMA 2020 flow diagram outlines the study selection process for this review (Fig. 1)^(48)^. A total of eleven articles were identified as eligible for review inclusion (Table 3).

**Table 3.** Studies investigating iodine among vegans, vegetarians and omnivores published after January 2020

Study, year	Study design	Location	Dietary groups	Sample		Method of dietary classification	Grouping adherence length	
				*N*	Female, male			
Abraham *et al.* 2022^(53)^	Cross-sectional	Berlin, Germany	Strict raw	16	5, 11	Classified according to RBVD study criteria. (not self-selected)	Diet for at least four months or at least one year	Raw food diet
			Vegan	32	18, 14			
			Omnivore	27	14, 13			
Blaurock *et al.* 2021^(54)^	Cross-sectional	Munich, Germany	Vegetarian	31	31, 0	Self-assessed	NA	
			Omnivore	30	30, 0			
Dawczynski *et al.* 2022^(49)^	Cross-sectional	Jena, Halle, and Leipzig, Germany	Vegan	58	41, 17	Self-assessed. Food intake data were also checked by researchers for discrepancies in diet selection	3·0	3·0*
			Vegetarian (lacto-ovo)	65	47, 18		6·0	10.*
			Flexitarian	70	56, 14		8·0	17·8*
			Omnivore	65	40, 25		32·0	20·0*
Eveleigh *et al.* 2022^(34)^	Cross-sectional	Nottingham, UK	Vegan (2016–17)	12	12, 0	Self-assessed. Food intake data were also checked by researchers for discrepancies in diet selection	> 6 months	
			Vegan (2019)	7	4,3			
			Vegetarian (including pescatarians) (2016–17)	5	5, 0			
			Vegetarian (including pescatarians) (2019)	10	9, 1			
			Omnivore (2016–17)	43	36, 7			
			Omnivore (2019)	19	14, 5			
Fallon and Dillon, 2020^(33)^	Cross-sectional	Lancashire, UK	Vegan	20	20, 0	The diaries were cross-checked to ensure they had correctly identified themselves	NA	
			Vegetarian	16	16, 0			
			Omnivore	26	26, 0			
García-Morant *et al.* 2020^(55)^	Cross-sectional	Spain	Vegan	102	67, 35	NA	NA	
			Omnivore	3323	1732, 1587			
Groufh-Jacobsen *et al.* 2020^(50)^	Cross-sectional	Oslo, Norway	Vegan	115	74, 41	Classified by researchers according to questions addressing animal product consumption	≥ 6 months	
			Vegetarian	55	43, 12			
			Pescatarian	35	31, 4			
Jakše *et al.* 2021^(56)^	Cross-sectional	Slovenia	Vegan	51	34, 17	Self-assessed. Food intake data were also checked by researchers for discrepancies in diet	Participants should have had the same (current) dietary pattern for ≥ 1 year	
			Omnivore (non-vegan)	29	16, 13			
Kowalska *et al.* 2020^(57)^	Data modelling	Poland	Vegan	Participants were only used for creation of the Omnivore (regular) diet. The other diets were generated by quantitative analysis of a total of 70 menus (10 for each diet type) prepared by dieticians		NA	NA	
			Vegetarian (lacto-ovo)					
			Pescatarian					
			Omnivore (fish free)					
			Omnivore (milk free)					
			Omnivore (basic)					
			Omnivore (regular)	30	12, 18			
Menzel *et al.* 2021^(51)^	Matched pairs (age and sex)	Germany	Vegan	36	18,18	Self-assessed	4·8	3·1–8·7*
			Omnivore	36	18,18			
Whitbread *et al.* 2021^(52)^	Cross-sectional	South Australia	Vegan	31	31, 0	Self-assessed	NA	
			Omnivore	26	26, 0			

NA, not assessed.

* Median and IQR.

### Urinary iodine status

**Fig. 2.**
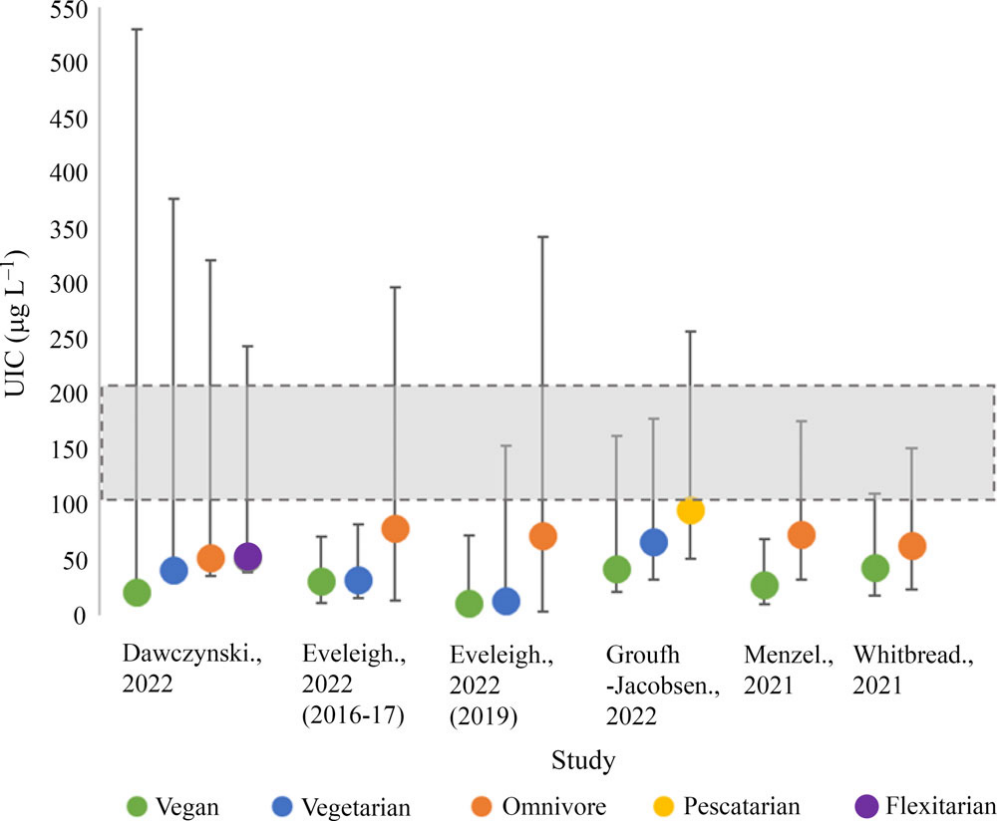
Iodine status measurements of included studies by median urinary iodine concentration (UIC). Shaded grey areas illustrate the WHO criteria for optimal iodine status (100–200 µg/l)^(11)^.

Iodine status by UIC was investigated in five studies with a total of 700 participants (vegan; 271, vegetarian; 135, pescatarian; 35, omnivore; 189, flexitarian; 70)^(34,49–52)^ (Fig. 2). Three studies assessed UIC using single-spot urine samples^(34,50,52)^; the remaining two studies used 24-h urinary samples^(49,51)^. We were unable to separate UIC estimates by sex, as all included studies provided either mixed-sex estimates or estimates in females alone.

Vegan participants had the lowest median UIC in all five studies (12·2–44·0 µg/l)^(34,49–52)^, with the lowest value recorded by Eveleigh *et al.* in UK vegans (12·2 µg/l)^(34)^. In all studies, vegans had significantly lower median UIC compared with omnivores (*P* < 0·05)^(34,49–52)^. Vegetarians in the included studies tended to have median UIC values greater than vegans but lower than omnivores, flexitarians and pescatarians^(49,50)^. Only one study reported the median UIC of vegetarians to be significantly lower than omnivores (*P* < 0·05)^(34)^. Pescatarians in Norway experienced the greatest median UIC of 96·0 µg/l^(50)^. The median UIC of flexitarians was reported in one study (52·0 µg/l) and was slightly lower than the omnivores in the same study (53·0 µg/l)^(49)^. The included studies showed considerable variation in UIC recorded within dietary groups, for example, the upper range for median UIC for vegans in Dawcynski *et al.* study was 509 µg/l^(49)^.

None of the dietary groups included in this review had median UIC values within the optimal range for iodine status (100–200 µg/l) according to WHO criteria and would be classified as iodine-deficient (mild–severe; 50–99 µg/l–> 20 µg/l)^(34,49–52)^. Severe iodine deficiency (> 20 µg/l) was observed in one study of vegans living in the UK^(34)^. Moderate deficiency (20–49 µg/l) was observed in all vegan diets and 75 % (3/4) of vegetarian diets^(34,49–52)^. Omnivores, flexitarians and pescatarians had median UIC values within the range of mild iodine deficiency (50–99 µg/l)^(34,49–52)^. None of the dietary groups in our current review had median UIC values relating to excessive iodine intake (> 300 µg/l)^(34,49–52)^.

None of the studies investigating iodine status used creatinine correction^(34,49–52)^.

### Dietary iodine intake

**Fig. 3.**
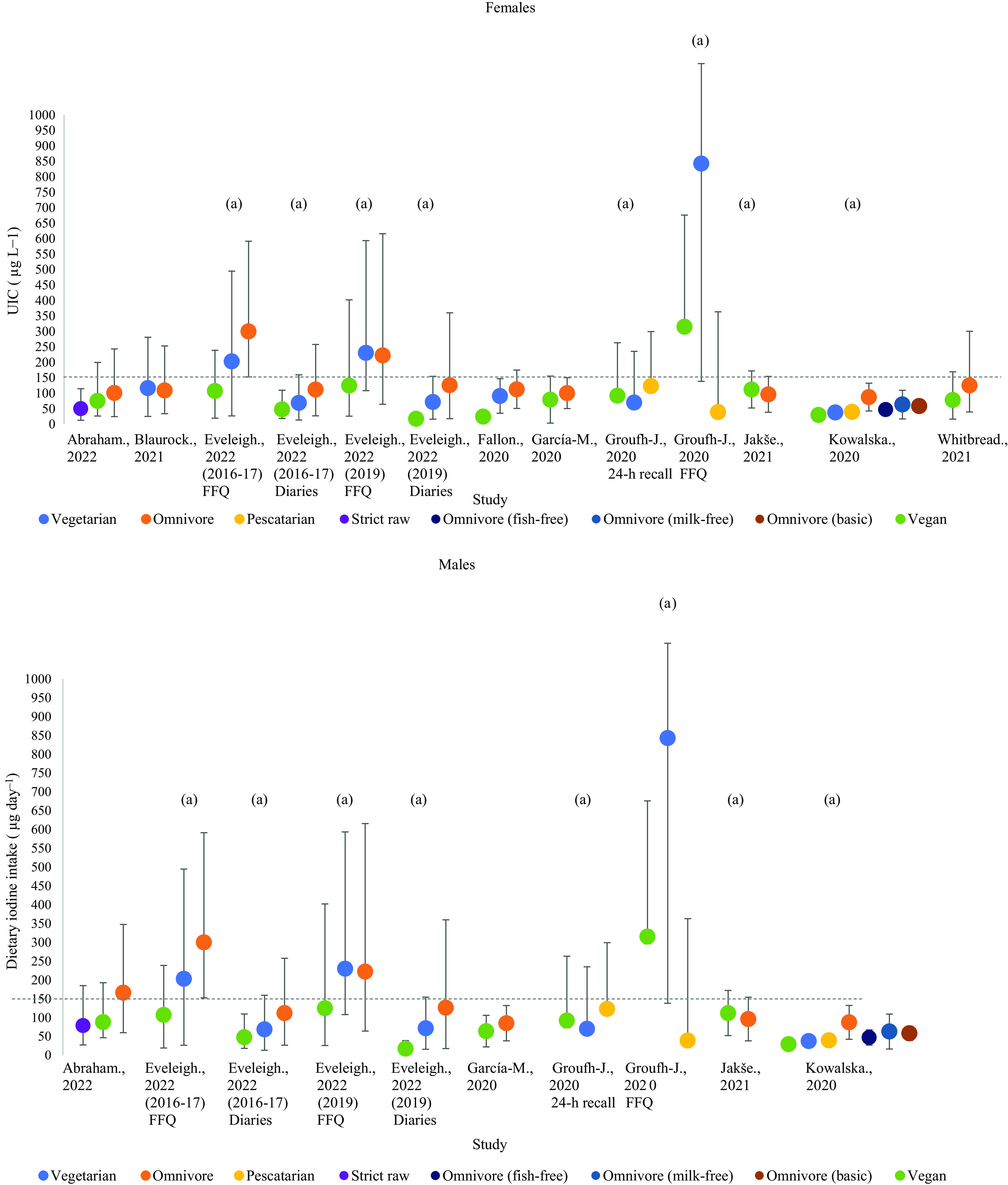
Visual representation of estimated average iodine intake (µg/d) for females and males in included studies. The grey dashed line represents the adequate intake recommended by the WHO of 150 µg/d^(11)^. (a) mixed-sex values. Significance values are not presented within this figure. See Table 4.

Dietary iodine intakes were recorded in nine studies and included 678 participants (strict raw; 16, vegan; 280, vegetarian; 117, pescatarian; 35, omnivore; 230; Table 4; Fig. 3)^(33,34,50,52–57)^. All studies evaluated iodine intake between vegans and one or more dietary groups^(33,34,50,52–57)^. Most studies reported mixed-sex estimates of dietary iodine. Only two studies separated dietary iodine intake by sex^(53,55)^, and three studies only recruited females to study^(33,52,54)^. Sixty-seven per cent of studies (6/9) were conducted in countries considered ‘adequate’ according to national data from the Global Scorecard of Iodine Nutrition (2021)^(20,33,34,52,55–57)^.

**Table 4.** Assessment of dietary iodine intake for vegans, vegetarians and omnivores in included studies

Study, year	Assessment of dietary iodine	Criteria for iodine intake	Dietary group		Dietary iodine intake (µg/d)		Contribution of iodised salt, seaweed, and iodine-containing supplements	Meeting criteria (Y/N)‡	National salt fortification status	National iodine intake from the Global Scorecard of Iodine Nutrition (2021)
			*N*	Female, male	Median/Mean	Q1 –Q3/sd				
Abraham *et al.* 2022^(53)^	3-d weighed food diaries	D-A-CH reference values: F:150 µg/d, M:180 µg/d	Strict raw 16	5, 11	F: 49·6	37·4–64·6*	Twelve participants (75 %) recorded intake of dietary supplements (B_12_ and vitamin D). The consumption of iodine-containing supplements and iodised salt was not stated. Only individuals in the strict raw diet group consumed seaweed, but the contribution to iodine was not discussed.	N	Voluntary	Insufficient
					M: 78·9	51·7–106·0*				
			Vegan 32	18, 14	F: 74·8	48·9–124·6*		N		
					M: 88·0	41·6–104·5*				
			Omnivore 27	14, 13	F: 100·5	76·5–142·6*		N		
					M: 165·9	106·2–181·5*				
Blaurock *et al.* 2021^(54)^	Self-administered, online 157 -item FFQ as part of the eNutri2019-DE web application analysed by Bundeslebensmittelschlüssel, BLS database	Dietary reference values (DRV) Germany 200 µg/d (18–51 years)	Vegetarian 31	31, 0	F: 116·7	92–164*	Seventy-four percent of vegetarians and 63 % of omnivores reported consuming dietary supplements. The consumption of iodine-containing supplements, iodised salt and seaweed was not stated.	N	Voluntary	Insufficient
			Omnivore 30	30, 0	F: 108·6	75–144*		N		
Eveleigh *et al.* 2022^(34)^	Online iodine-specific 45-item FFQ and 3-d food diaries analysed by a dietary software program, Nutritics	Reference Nutrient Intake (RNI) for UK 140 µg/d		Vegan (2016–17) FFQ: 12 FD: 10	FFQ: 107·0	88·0–131·5*	Multivitamins containing iodine were consumed by 12 % of participants providing an average of 140 µg/d to dietary iodine. Multivitamin supplements provided significantly greater iodine to the diets of vegans compared with omnivores and vegetarians (*P* = < 0·001 and *P* = 0·033, respectively). Seaweed supplements kelp and/or kombu were consumed by two omnivores in 2019, providing an average iodine intake estimate of 1541 µg/d. Seaweed supplied a significantly greater iodine contribution to the diets of vegans compared with omnivores (*P* = 0·040). One omnivore in 2016–17 recorded consuming iodised salt contributing 61 % of dietary iodine.	FFQ: N Food diaries: N	None	Adequate
					Food diaries: 47·6	29·6–61·8*				
				Vegetarian (2016–17) FFQ: 5 FD: 4	FFQ: 202·9	176·5–291·7*		FFQ: Y Food diaries: N		
					Food diaries: 68·8	55·8–90·4*				
				Omnivore (2016–17) FFQ: 34 FD: 27	FFQ: 299·6	147·2–291·7*		FFQ: Y Food diaries: N		
					Food diaries: 111·9	85·4–145·6*				
				Vegan (2019) FFQ: 7 FD: 4	FFQ: 124·9	99·3–277·0*		FFQ: N Food diaries: N		
					Food diaries: 17·3	17·2–21·4*				
				Vegetarian (2019) FFQ: 9 FD: 6	FFQ: 230·0	122·0–363·4*		FFQ: Y Food diaries: N		
					Food diaries: 71·4	55·7–82·8*				
				Omnivore (2019) FFQ: 19 FD: 11	FFQ: 221·9	157·9–393·7*		FFQ: Y Food diaries: N		
					Food diaries: 126·0	108·5–233·8*				
Fallon and Dillon, 2020^(33)^	4-d food diaries analysed by a dietary software program, Nutritics	RNI for UK 140 µg/d and DRV 150 µg/d.	Vegan 20	(20, 0)	F: 24·4	12·7†	Seaweed, salt or supplements not measured.	N	None	Adequate
			Vegetarian 16	(16, 0)	F: 90·8	55·9†		N		
			Omnivore 26	(26, 0)	F: 112·6	62·1†		N		
García-Morant *et al.* 2020^(55)^	Online 24-h food recalls analysed by a dietary software program, DIAL and data from the Spanish Agency for Food Safety and Nutrition National Dietary Intake Survey	None provided	Vegan 102	(67, 35)	F: 79·0	76·0†	Dietary supplements were recorded and combined with dietary intake data. Seaweed and iodised salt not measured.	N	Voluntary	Adequate
					M: 64·0	42·0†				
			Omnivore 3321	(1589, 1732)	F: 100·0	50·0†		N		
					M: 85·0	47·0†				
Groufh-Jacobsen *et al.* 2020^(50)^	24-h food recalls and thirty-two-item FFQ updated from the Norwegian Food Composition Table 2019	Estimated Average Requirement (EAR) of 100 µg/d	Vegan 115	(74, 41)	24-h recall: 92·0	19·0, 171·0*	The median contribution of supplements to iodine intake of 24-h recalls were 150 µg/d for vegans (*n* 57), vegetarians (*n* 25) and pescatarians (*n* 18). For FFQ, estimates supplements also contributed a median average of 150 µg/d to dietary iodine intake of vegans (*n* 69), vegetarians (*n* 26) and pescatarians (*n* 10). Seaweed (microalgae) provided 865 µg/d (364, 1978) to vegans (*n* 23), 843 µg/d (705, 1590) to vegetarians (*n* 8) and 375 µg/d (110, 610) to pescatarians (*n* 4). Iodised salt not measured.	24-h recall: N FFQ: Y	Voluntary	Insufficient
					FFQ: 315·0	19·0, 361·0*				
			Vegetarian 55	(43, 12)	24-h recall: 70·0	17·0, 165·0*		24-h recall: N FFQ: Y		
					FFQ: 843·0	705·0, 1590·0*				
			Pescatarian 35	(31, 4)	24 h: 123·0	16·0, 176·0*		24-h recall: Y FFQ: Y		
					FFQ: 39·0	16·0, 324·0*				
Jakše *et al.* 2021^(56)^	52-item FFQ analysed by a dietary software program, Open Platform for Clinical Nutrition (OPEN)	D-A-CH reference values, F:150 µg/d, M:180 µg/d	Vegan 51	34, 17	112·0	60·0†	Dietary supplements were recorded and combined with dietary intake data. Dietary supplements provided > 25 % of total iodine intake for vegans and omnivores (non-vegans) Seaweed and iodised salt not measured.	N	Mandatory	Adequate
			Omnivore (non-vegan) 29	16, 13	96·0	58·0†		N		
Kowalska *et al.* 2020^(57)^	Modelled diets were created by trained dieticians	EAR for Poland 95 ug/d		Vegan	29·4	10·3†	Seaweed, salt, or supplements not measured.	N	Mandatory	Adequate
				Vegetarian (lacto-ovo)	37·6	9·2†		N		
				Pescatarian	39·2	11·1†		N		
				Omnivore (fish-free)	46·9	20·3^2^		N		
				Omnivore (milk-free)	62·8	46·4^2^		N		
				Omnivore (basic)	57·9	19·1†		N		
				Omnivore (regular)	87·2	45·0†		N		
Whitbread *et al.* 2021^(52)^	24-h diet food recalls by a smartphone app and food diaries analysed by a dietary software program, FoodWorks Dietary Analysis package	Recommended daily intake (RDI) 150 ug/d, EAR 100 ug/d and Nutrient Reference Values (NRV) for Australia and New Zealand (NHMRC)	Vegan 31	31, 0	78·0	62·0–91·0*	Participants who frequently consumed iodine-containing supplements were excluded. Six vegans reported ingesting seaweed (2·7 g/d), but iodine intake from seaweed was recorded as 0·0 ug/d. Iodised bread was the main iodised food source in vegans. Iodised salt was consumed by four vegan and four omnivore participants.	N	Mandatory	Adequate
			Omnivore 26	26, 0	125·0	86·0–175·0*		N		

* Median (Q1–Q3).

† Mean ± sd.

‡ According to WHO criteria of 150 ug/d.

Food diaries > 3 d (44 %, 4/9) and FFQ (44 %, 4/9) were the most commonly used dietary method to measure iodine intake^(33,34,53,54,56)^. Other dietary methods included 24-h food recalls (33 %, 3/9)^(50,52,55)^. Three studies addressed iodine intake using two dietary methods to assess both short-term and longer-term dietary intake^(34,50,52)^.

Vegan participants living in the UK in 2019 had the lowest average iodine intake of 17·3 µg/d (17·2–21·4 µg/d; mixed-sex estimates)^(34)^. The greatest iodine intake was recorded in vegetarians in Oslo, Norway, at 843·0 µg/d (705·0–1590·0 µg/d; mixed-sex estimates)^(50)^. Omnivores had the greatest dietary iodine intake in 70 % (7/10) of included cohorts^(33,34,52,53,55,57)^, whereas vegans had the lowest iodine intake in 73 % of cohorts (8/11)^(33,34,52,55,57)^. There was substantial variation in iodine intake within dietary groups with the greatest variation being observed by Groufh-Jacobsen *et al* in FFQ estimates of pescatarians (705·0–1590·0 µg/d)^(50)^.

Seven studies recorded a significant difference in iodine intake between one or more dietary group (*P* = < 0·05)^(33,34,50,52,53,55,56)^. Four studies found the iodine intake of vegans to be significantly lower than omnivores (*P* = < 0·001)^(33,34,52,55)^. Vegans and vegetarians had significantly lower habitual iodine intake compared with pescatarians in one study^(50)^. Raw food eaters were recorded to have significantly lower iodine intake than vegans and omnivores^(53)^. The study conducted by Kowalska *et al.* (2020) did not statistically evaluate the difference in iodine intake between groups. There were differences in significance between groups according to methods of assessing iodine intake. In the study by Groufh-Jacobsen *et al.* (2020), a significant difference in iodine intake between vegans, vegetarians and pescatarians was only observed in FFQ estimates and not 24-h recalls. However, Eveleigh *et al.* did not observe a significant difference in iodine intake by FFQ in their 2019 cohort but did see differences when measured using food diaries^(34)^.

The WHO recommended iodine intake of 150 µg/d was used for the assessment of adequate dietary intake^(11)^. None of the included studies recorded estimates above the adequate range for all dietary groups. Iodine inadequacy was reported in all dietary groups in 75 % (9/12) of the cohorts assessed^(33,34,50,52–57)^. Only two studies reported at least one dietary group to have adequate average iodine intake^(34,50)^.

Five studies addressed the types of foods consumed by different dietary groups^(34,52–54,56)^, of which three estimated the possible contribution of specific food groups to total iodine intake^(34,50,52)^. Overall, the vegan and vegetarian groups consumed greater quantities of plant-based food groups (fruit, vegetables, legumes, tubers, cereals and grains)^(34,52–54,56)^. Two studies recorded alternative milk intake to be significantly greater in vegan and vegetarian groups^(34,52)^. Within these two studies, dairy products and eggs were the greatest source of iodine for individuals not restricting these foods. Kowalska *et al.* (2020) created model diets for each dietary group and used foods that were typically consumed according to dieticians’ advice and current research^(57)^. Foods present in the models of vegan diets included cereal, fruit, vegetables, nuts, mushrooms, legumes, oils and alternative milk.

Mandatory or voluntary USI was present in seven of the included studies, and investigations conducted in the UK had no USI programme^(33,34)^. Only two studies addressed the relative consumption of iodised salt to total iodine intake^(34,52)^. Iodised salt was only consumed by vegan (*n* 4; 4/31)^(52)^ and omnivorous participants (*n* 5; 1/34 & 4/26)^(34,52)^. Whitbread *et al.* (2021) was the only study to monitor the intake of bread fortified with iodine, an average quantity of 28 g/d and 36 g/d for vegans and omnivores consumed^(52)^. Consumption of iodised bread was similar between dietary groups and was acknowledged as a good source of iodine. The consumption of iodine-containing supplements to total iodine intake was recorded in five studies^(34,50,54–56)^. Two of the studies that reported supplement use did not provide data on the actual contribution of iodine-containing supplements to total intake^(53,54)^. One study prevented supplement intake during the study^(52)^. Four studies addressed the consumption of seaweed and microalgae in different dietary groups^(34,50,52,54)^; however, in two of these studies, the relative contribution of seaweed to iodine was not discussed^(52,53)^. Vegans (*n* 29) were most likely to consume seaweed as part of their diet. Seaweed intake was also reported in strict raw food eaters (*n* 1), vegetarians (*n* 8), pescatarians (*n* 4) and omnivores (*n* 2)^(34,50,52,53)^.

#### Meta-analysis of iodine status and intake in vegans and vegetarians compared with omnivores

**Fig. 4.**
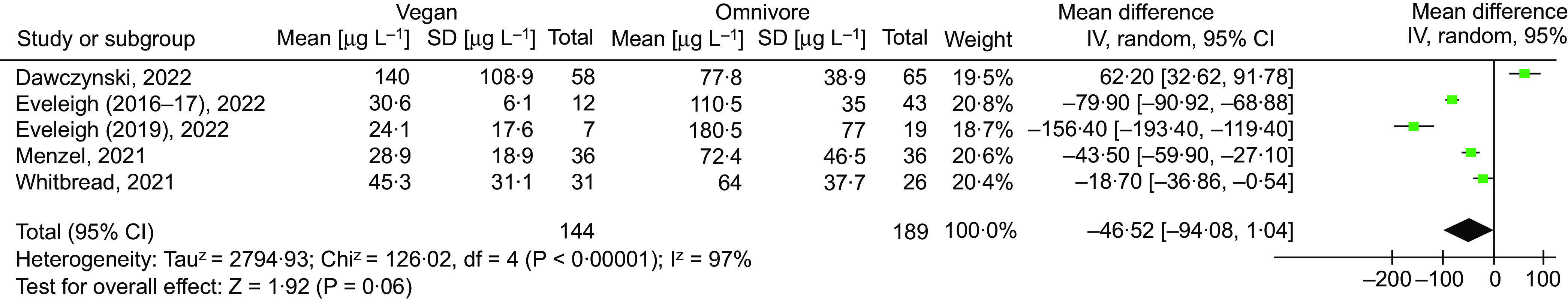
Meta-analysis forest plot comparing the effect of a vegan *v*. omnivorous diet on iodine status. Data presented is female and mixed-sex estimates only.

**Fig. 5.**
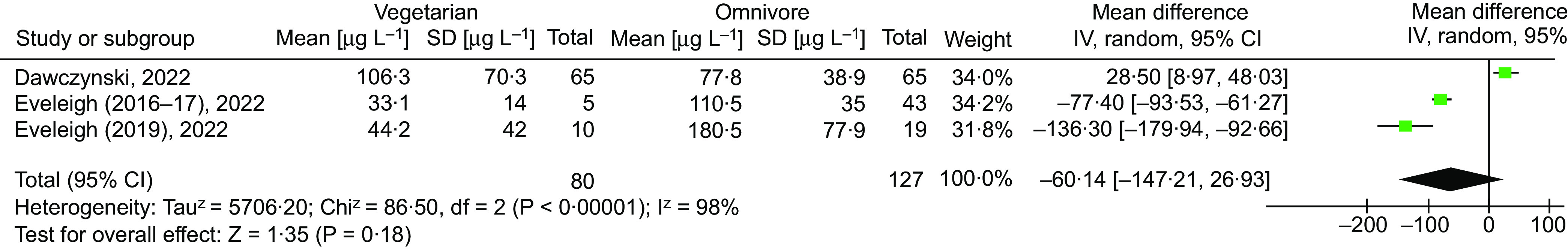
Meta-analysis forest plot comparing the effect of a vegetarian *v*. omnivorous diet on iodine status. Data presented is female and mixed-sex estimates only.

**Fig. 6.**
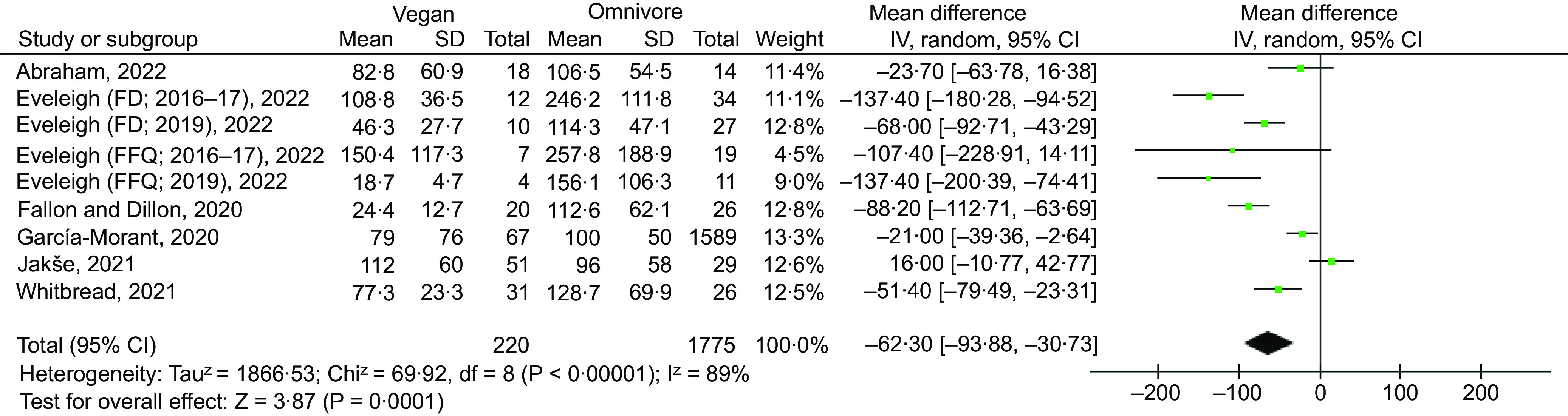
Meta-analysis forest plot comparing the effect of vegan *v*. omnivorous diet on iodine intake. Data presented is female and mixed-sex estimates only.

**Fig. 7.**
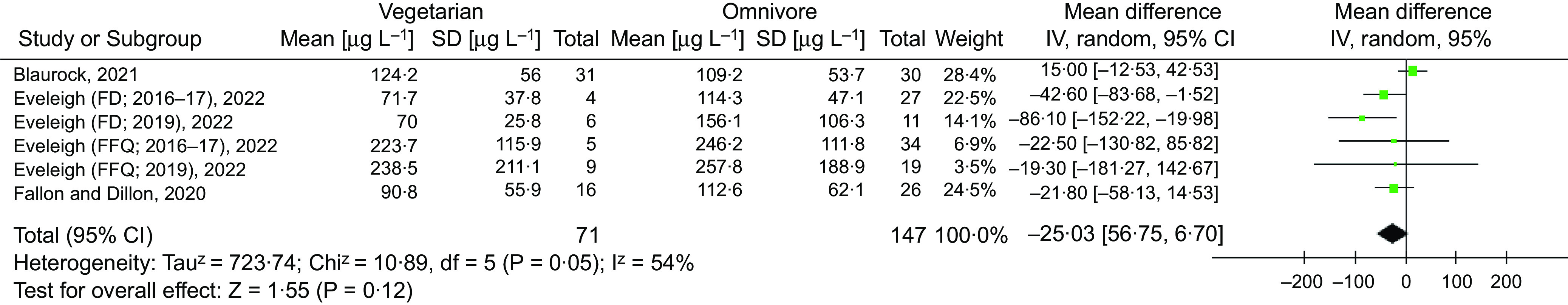
Meta-analysis forest plot comparing the effect of vegetarian *v*. omnivorous diet on iodine intake. Data presented is female and mixed-sex estimates only.

There was a strong trend for vegan or vegetarian diets to be associated with reduced iodine status as measured by UIC, but this did not quite reach significance (Fig. 4 and 5; *P* = 0·06 & *P* = 0·18), so no subgroup analysis was completed. There was, however, an overall significant negative effect of vegan diets on iodine intake (*P* = < 0·001; Fig. 6), with no effect observed for vegetarian diets (*P* = 0·12; Fig. 7). Subgroup analysis for iodine intake vegan *v* omnivore is shown in Supplementary Fig. 1. Significant effects were shown for female-only intake (*P* = 0·007), mixed-sex intake (*P* = 0·01), living in a country with voluntary (*P* = 0·01) or no USI programme (*P* = *P* < 0·001), and living in a country with national iodine intake considered as adequate (*P* = <0·001). In countries with mandatory salt iodisation, there was no effect of vegan diet on iodine intake (*P* = 0·60).

## Discussion

This systematic review is an update of our previous work, investigating the evidence for iodine intake and status among adults following vegan and vegetarian diets, to reflect dietary consumption in the modern day. The publication of eleven studies eligible for inclusion in the past 2 years since our previous review demonstrates that there is an increased interest and awareness in this area. Our systematic review confirms that individuals following vegan and vegetarian diets, living in countries without mandatory salt iodisation, and not consuming seaweed or iodine-containing supplements, still have an increased risk of low iodine status, iodine deficiency, and inadequate iodine intake compared with less restrictive dietary groups^(28)^. Our review also highlights that iodine nutrition is poor in many subgroups of the global population and that difficulties achieving iodine recommendations are not unique to those following restrictive diets.

In our previous review^(28)^, we found that the degree of vulnerability in all dietary groups appeared to be impacted by the standing of national iodine estimates, whereby the median UIC of omnivorous participants studied tended to reflect a country’s national data. In our present review, we found that on average urine samples provided by omnivorous participants were lower in iodine than national estimates^(34,49–52)^. Only in studies conducted in Germany were omnivores within the same bracket of the WHO criteria as national data (89 μg/l; 50–99 μg/l mild)^(11,20,49,51)^. However, there is still a general trend in which vegans are likely to have median UIC corresponding to a lower bracket of the WHO criteria than omnivores recruited in the same study^(34,49–52)^. Although, the median UIC of all dietary groups studied in this review fell below the WHO criteria for optimal iodine status (100–200 µg/l)^(11,34,49–52)^.

We found that 67 % (6/9) of studies investigating iodine intake were conducted in countries considered ‘adequate’ according to national data from the Global Scorecard of Iodine Nutrition (2021)^(33,34,52,55–57)^. But, none of the included studies recorded estimates above the adequate range for all dietary groups^(33,34,50,52–57)^. Equally, iodine inadequacy was reported in 75 % (9/12) of the cohorts assessed^(33,34,50,52–57)^. The global scorecard population monitoring uses the WHO recommendation of median UIC from school-aged children or women of childbearing age as a proxy for assessing adequate iodine intake on a national level^(20)^. The use of median UIC from school-aged children as an indicator of iodine intake has been criticised for masking particular subgroups with varying diets and/or iodine sources^(19)^. Women are a subgroup more vulnerable to the consequences of iodine deficiency and are twice as likely as men to adopt a vegan or vegetarian diet^(58)^. Three studies assessed iodine intake specifically in women of childbearing age^(33,52,54)^. Iodine intake was found to be below the WHO recommendation of 150 µg/d in all studies of women in our review regardless of dietary choice^(33,52,54)^. Moderate iodine deficiency pre-conception increases the risk of infant mortality by stillbirth and miscarriage^(59,60)^. Furthermore, iodine deficiency *in utero* may disrupt normal fetal neurodevelopment and cause irreversible neurological damage^(59,60)^. Our present review highlights that iodine intake must be assessed among different subsets of the population to identify groups at risk of deficiency.

Vegan participants living in the UK were recorded to have the lowest average iodine intake of 17·3 µg/d and 24·4 µg/^(33,34)^. Dietary intake estimates recorded by Eveleigh *et al* (2022) and Fallon *et al.* (2020) were much lower than past data collected on British vegans^(32,61,62)^, suggesting that iodine intake has reduced over time. Vegans living in the UK may find it more challenging to meet their iodine requirements because along with restricting rich sources of iodine from animal sources, individuals selecting these diets also live in a country with no current USI programme. Salt iodisation is an effective and sustainable way of improving iodine intake^(19)^. Currently, 145 countries globally have enrolled in either mandatory (124/145) or voluntary (21/145) USI^(19)^. Apart from the UK, all other included studies were conducted in a country with USI^(50,52–57)^. Iodised salt can be purchased in the UK, but its availability is low and tends to be more expensive than non-iodised household varieties^(63)^. Eveleigh *et al* (2022) found that only one omnivorous participant recorded consuming iodised salt equating to 61 % of their total dietary iodine. However, it is very difficult to quantify the amount of salt added to meals and used or lost during cooking^(19)^. Additionally, the iodine content of fortified salt can vary considerably^(19)^. Whitbread *et al* (2021) was the only study, conducted in a location with USI, to estimate the relative contribution of iodised salt and foods containing iodised salt such as bread^(52)^. Hence, dietary iodine intake may be lower in many of the included studies because the involvement of iodised salt was not estimated.

Bread has been selected as a vehicle for iodine fortification in countries, including Australia, New Zealand (NZ) and Denmark^(52)^. In most countries, bread is a staple food product that can be consumed by nearly everyone in the population including those following vegan and/or vegetarian diets making it a practical method of iodine fortification. In the study by Whitbread *et al* (2021), iodised bread was the main iodised food source in the diets of Australian vegans and greater bread intake (g/d) correlated significantly with improved UIC^(52)^. The mandatory fortification of bread in Australia is estimated to be about 53–70 μg per 100 g^(64)^. The British Dietetics Association (BDA) denotes a portion of bread is equivalent to one slice or 34–36 g^(65)^, if bread was fortified in the UK to the same level as in Australia, one portion would provide an average of 22 μg which could potentially boost iodine intake in all dietary groups.

Alternative milk has also been identified and promoted as a possible vehicle for iodine fortification for vegans and/or those who do not consume cows’ milk^(66)^. A recent study investigating the iodine fortification status of plant-based dairy and fish alternatives in the UK found that only 28 % of milk alternatives and 6 % of yogurt alternatives were fortified with iodine^(36)^. Similarly, no cheese or fish alternatives were found to be currently fortified with iodine^(36)^. Other studies have identified that individuals who consume alternative milk exclusively tend to have significantly lower iodine intake than cows’ milk consumers (94 *v*. 129 µg/d)^(37)^. Two studies in our review recorded alternative milk intake to be significantly greater in vegan and vegetarian groups^(34,52)^, and the displacement of cows’ milk may have contributed to lower iodine intake. In May 2021, the BDA’s England Board and the Maternal & Fertility Nutrition Specialist Group completed a project to consider the case for the wider iodine fortification of alternative milk to improve iodine intake^(67)^. However, at present, no UK policy has been implemented for the mandatory iodine fortification of any foods; therefore, individuals following a vegan and/or vegetarian diet are responsible for planning their diet to include appropriate sources of iodine (e.g. iodine supplements, occasional seaweed consumption and/or iodine fortified alternative milk).

Although mandatory USI measures are considered to be the optimal approach, most countries with voluntary fortification are iodine-sufficient^(19)^. We identified that individuals following a vegan diet living in countries with no mandatory USI programme were significantly more likely to have lower iodine intake. Our findings match the global experience; in that voluntary fortification may not benefit all subgroups of the population^(68)^. There is an urgent need to investigate the usefulness of mandatory iodine fortification to address the emergence of iodine deficiency in those selecting vegan or vegetarian diets.

The modelling study conducted by Kowalska *et al.* (2020) in Poland demonstrates the difficulty in achieving adequate iodine intake in a number of different diet types if iodine-fortified foods (including salt) or supplements are not available. In this study, modelled diet menus of vegan, vegetarian, pescatarian and variations of the omnivorous diet (fish free, milk free, basic and regular) were prepared by qualified dieticians with the use of methods avoiding nutritional deficiencies. Even though these diets had been appropriately planned by dieticians, all of the diets were characterised by insufficient iodine intake. In Poland, the main sources of dietary iodine are iodised salt and white fish including cod and pollock^(57)^. Therefore, it is unsurprising that iodine intake was observed to be lowest in individuals following diets that exclude fish (vegans, vegetarians and fish-free omnivores) when contribution of iodised salt was not added to intake estimates.

The greatest average iodine intake and status were recorded in Norwegian pescatarians (123·0 µg/d and UIC of 96·0 µg/l)^(50)^. Pescatarians do not consume meat but may eat fish, milk or eggs in varying quantities^(5)^. In a typical Norwegian diet, lean fish like cod, haddock and saithe are the richest sources of iodine along with cows’ milk and dairy products^(50)^. However, the iodine content of fish can vary significantly between species and geographical locations^(69)^. The Norwegian Directorate of Health recommends one portion of fish two or three times a week; however, annual fish intake has dropped in Norway across all age groups apart from the elderly; this decline is particularly noticeable in young people (18–34 years of age)^(70)^. The authors suggest that greater iodine intake in pescatarians in this study was more due to consumption of iodine-containing supplements rather than that of fish^(50)^, as this significantly improves iodine status^(71,72)^. A larger proportion of vegans and vegetarians consumed dietary supplements (inclusive of non-iodine-containing varieties) compared with omnivores, and in two studies, iodine-containing supplements provided between 140 and 150 µg/d to total iodine^(34,50)^. However, despite their consumption, the majority of participants had inadequate UIC and below-recommended iodine intake. The WHO recommends a daily iodine supplement dose of 150 µg/d for women of childbearing age (15–19 years) living in areas with insufficient access to iodised salt or vulnerable groups^(11)^. Considering the vulnerability of individuals in the included studies, the effect of iodine-containing supplements in vegan and vegetarian populations warrants further investigation.

We found previously that the greatest iodine intake was recorded for females following vegan diets, living in London (1448·0 ± 3879·0 µg/d), whose regular consumption of seaweed increased intakes to over six times the RNI^(62)^. In our present review, four studies addressed the consumption of seaweed and microalgae in different dietary groups^(34,50,52,53)^; however, not all studies considered their contribution to total iodine. Vegans were the most frequent consumers of seaweed which provided iodine intake close to the maximum tolerable level of 1000 µg/d. The iodine content of seaweed is high and varies considerably according to the species consumed^(25,26)^, with kombu having the greatest iodine content (2523·5 mg/kg) and so is not recommended to improve iodine intake due to the significant risk of excess^(25,26)^.

In our previous review, we discussed key issues with the methodology selected to record intake from vegan and vegetarian groups including accurately defining diets^(28)^, the use of outdated food tables or databases, and variation in techniques used to measure iodine in the diet (e.g. 24-h food recalls *v.* FFQ). These issues are still relevant to the present review and may reduce the accuracy of dietary intake estimates. Further limitations of this study include the relatively small number of included studies, oversimplifying of dietary practice to enable comparison of studies and a lack of studies that were well populated or well represent the general public.

### Conclusions

This review agrees with findings from our previous systematic assessment on this topic^(28)^. We found that vegans and vegetarians consuming seaweed or iodine-containing supplements continue to have increased risk of low iodine status, iodine deficiency and inadequate iodine intake compared with adults following less restrictive diets living in countries lacking mandatory iodisation of salt. Similarly to our previous review, there is a relationship between national iodine deficiency and the degree of vulnerability to vegans and vegetarians. However, we also conclude that iodine nutrition is inadequate in many subgroups, and that complications achieving iodine recommendations are universal. There is an urgent need to monitor iodine intake and status in at risk populations including young women and those following a vegan or vegetarian diet. In addition to research into safe routes of improving iodine intake in vegan and vegetarian populations living in regions where staple foods are not fortified or USI coverage is not present or is not mandatory. Further awareness of how to appropriately plan a vegan or vegetarian diet to achieve iodine recommendations is required.

## Supplementary material

For supplementary material accompanying this paper visit https://doi.org/10.1017/S000711452300051X. click here to view supplementary material
