# Supplementary material for: Systematic review and meta-analysis of iodine nutrition in modern vegan and vegetarian diets
Source: Br J Nutr. 2023 Mar 13;130(9):1580–94. doi: 10.1017/S000711452300051X (PMC10551477; doi:10.1017/S000711452300051X)
Supplement: Supplementary file 1 [file S000711452300051Xsup.zip › S000711452300051Xsup002.docx]

**Supplementary Figure 1.** Meta-analysis sub-group analysis forest plot comparing the effect of vegetarian v. omnivorous diet on iodine intake. Data presented is female and mixed gender estimates only. USI; Universal salt iodisation. 95% CI; confidence interval

| **Supplementary Table 1.** Search terms used in the study | |
| --- | --- |
| 1 | exp Iodine/ |
| 2 | Iodine*.mp |
| 3 | Iodine* Status* |
| 4 | Iodine* Intake* |
| 5 | Exp Deficiency |
| 6 | Exp Hypothyroidism |
| 7 | Vegetarian*.mp. |
| 8 | Exp Diet, Vegetarian/ |
| 9 | Vegan*.mp. |
| 10 | Exp Diet, Vegan/ |
| 12 | Fruitarian*.mp. |
| 13 | Flexatarian*.mp. |
| 14 | Pescatarian*.mp |
| 15 | Herbivore*.mp. |
| 16 | Plant?Based*.mp. |
| 17 | 7 OR 8 OR 9 OR 10 OR 11 OR 12 OR 13 OR 14 OR 15 |
| 18 | 16 AND 17 |
| Limit all to ‘Humans’, ‘Adult (18 years)’ and ‘English Language’ | |

| **Supplementary Table 2.** Quality assessment of included Observational Cohort, Cross-Sectional, and Case-Control studies using the Newcastle-Ottawa scale | | | | |
| --- | --- | --- | --- | --- |
| **Study, year** | **Selection**  **(max 5 stars)** | **Comparability**  **(max 2 stars)** | **Outcome**  **Assessment (or Exposure for Case-Control studies)**  **(max 3 stars)** | **Quality score^1^** |
| Abraham *et al*, 2022 | ★★★ | ★ | ★★ | Good |
| Blaurock *et al*, 2021 | ★★ | ★ | ★★ | Fair |
| Dawczynski *et al*, 2022 | ★★★★ | ★ | ★★ | Good |
| Eveleigh *et al*, 2022 | ★★★ | ★ | ★★ | Good |
| Fallon and Dillon, 2020 | ★★★ | ★ | ★★ | Good |
| García-Morant *et al*, 2020 | ★★ | ★ | ★★ | Fair |
| Groufh-Jacobsen *et al*, 2020 | ★★★★ | ★ | ★★ | Good |
| Jakše et al, 2021 | ★★★ | ★ | ★★ | Good |
| Menzel et al, 2021^2^ | ★★★ | ★★ | ★★ | Good |
| Whitbread et al, 2021 | ★★★ | ★ | ★★ | Good |
| ^1^ Quality score: Good = 3 or 4 stars in ‘selection’ and 1 or 2 stars in ‘comparability’ and 2 or 3 stars  in ‘outcome/exposure’; Fair = 2 stars in ‘selection’ and 1 or 2 stars in’ comparability’ and 2 or 3  stars in ‘outcome/exposure’; Poor = 0 or 1 star in ‘selection’ OR 0 stars in ‘comparability’ OR 0 or 1  stars in ‘outcome/exposure’  ^2^ Quality assessment has been conducted by the Newcastle-Ottawa scale for Case-Control studies | | | | |
