# Supplementary figures and images for: Systematic review and meta-analysis of iodine nutrition in modern vegan and vegetarian diets
Source: Br J Nutr. 2023 Mar 13;130(9):1580–94. doi: 10.1017/S000711452300051X (PMC10551477; doi:10.1017/S000711452300051X)

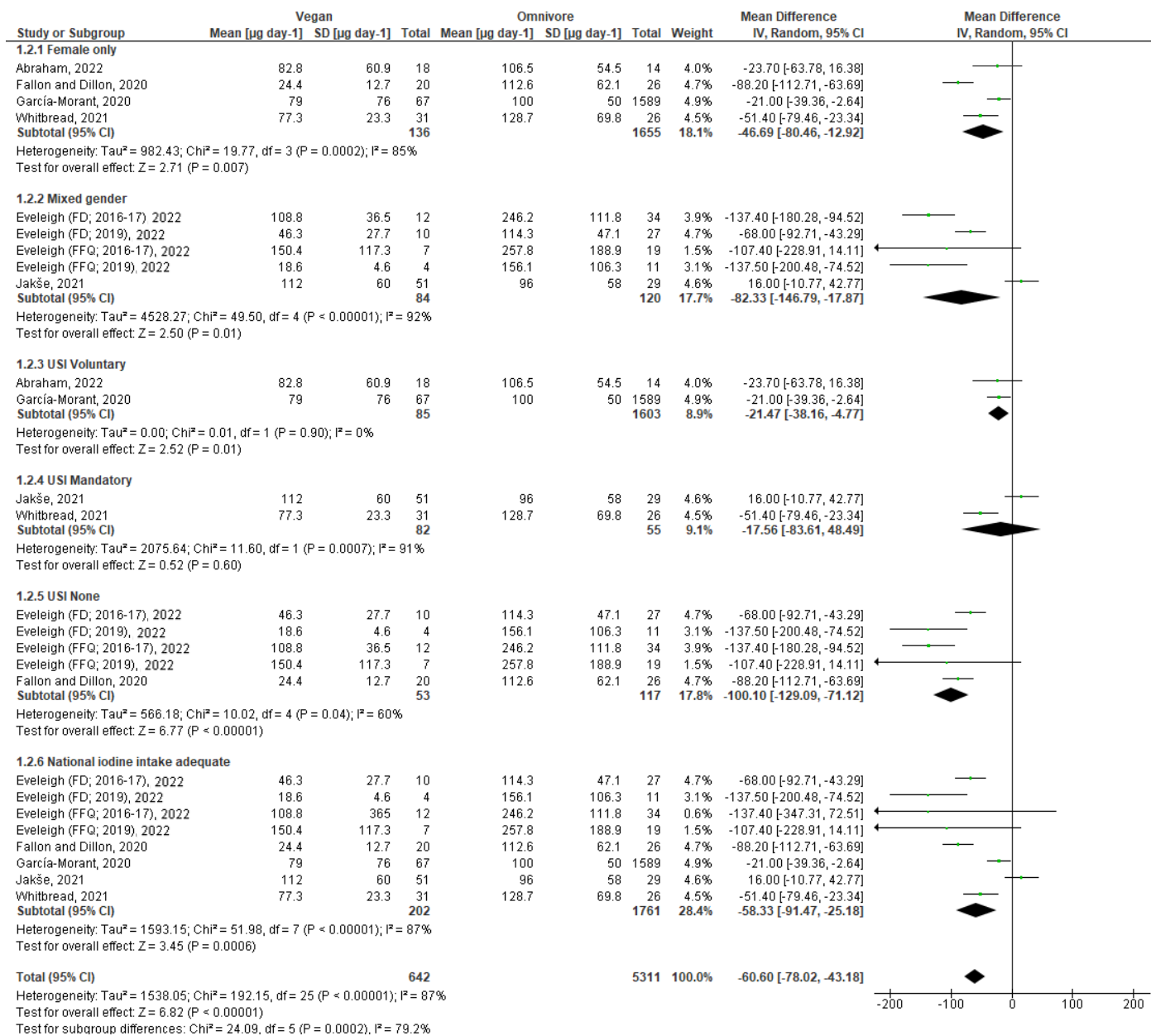

Supplement: Supplementary file 1 [file S000711452300051Xsup.zip › S000711452300051Xsup001.pdf]
